# A novel nomogram and risk classification system predicting the Ewing sarcoma: a population-based study

**DOI:** 10.1038/s41598-022-11827-z

**Published:** 2022-05-17

**Authors:** Yongshun Zheng, Jinsen Lu, Ziqiang Shuai, Zuomeng Wu, Yeben Qian

**Affiliations:** 1 Department of General Surgery, The First Affiliated Hospital of Anhui Medical University, 218 Jixi Road, Hefei, 230022 Anhui China; 2 Department of Orthopedics, The First Affiliated Hospital of University of Science and Technology of China, 17 Lujiang Road, Hefei, 230001 Anhui China; 3 Department of Orthopedics, The First Affiliated Hospital of Anhui Medical University, 218 Jixi Road, Hefei, 230022 Anhui China

**Keywords:** Computational biology and bioinformatics, Oncology, Risk factors

## Abstract

Ewing sarcoma (ES) is a rare disease that lacks a prognostic prediction model. This study aims to develop a nomogram and risk classification system for estimating the probability of overall survival (OS) of patients with ES. The clinicopathological data of ES were collected from the Surveillance, Epidemiology and Final Results (SEER) database from 2010 to 2018. The primary cohort was randomly assigned to the training set and the validation set. Univariate and multiple Cox proportional hazard analyses based on the training set were performed to identify independent prognostic factors. A nomogram was established to generate individualized predictions of 3- and 5-year OS and evaluated by the concordance index (C-index), the receiver operating characteristic curve (ROC), the calibration curve, the integrated discrimination improvement (IDI) and the net reclassification improvement (NRI). Based on the scores calculated with the nomogram, ES patients were divided into three risk groups to predict their survival. A total of 935 patients were identified, and a nomogram consisting of 6 variables was established. The model provided better C-indices of OS (0.788). The validity of the Cox model assumptions was evaluated through the Schönfeld test and deviance residual. The ROC, calibration curve, IDI and NRI indicated that the nomogram exhibited good performance. A risk classification system was built to classify the risk group of ES patients. The nomogram compares favourably and accurately to the traditional SEER tumour staging systems, and risk stratification provides a more convenient and effective tool for clinicians to optimize treatment options.

## Introduction

Ewing sarcoma (ES) is a primary malignant bone tumour composed of proliferating undifferentiated small round cells^1^. As the third most common malignant bone tumour after osteosarcoma and chondrosarcoma, the incidence of ES begins to increase in the second decade of life^2^. Additionally, ES is more common in Caucasian populations and has a slight male predominance (sex ratio of 3:2)^3,4^. It has been reported that people of African and Asian descent had lower incidence rates (0.2 and 0.8 cases per million, respectively), whereas Pacific Islanders, North African/Middle Eastern and European had higher incidence rates, and the incidence rate of European was as high as 1.5 cases per million^4,5^. The disease most often presents adjacent to bone, and a quarter arises in soft tissues^6,7^. The most common site for ES was found on long bones, especially the femur, tibia and humerus, and the recurrence and mortality rates of long bone ES were the second highest among all ES locations, following pelvic ES^8^. The median overall survival of ES patients is less than 12 months^9^. Before chemotherapy was introduced, only 10% of ES patients survived^10^. Due to the progress of multimodal treatments such as surgery, chemotherapy and radiotherapy, the 5-year survival rate of local ES that responds to multimodal therapy has increased to 55–65%^11^. In contrast, the survival rate of patients with metastases is less than 30% for 5 years^12^. The lungs (50%), bone (25%), and bone marrow (20%) are the most common sites for metastases, followed by the liver and brain^13^. Due to the rarity of ES, its prognostic factors are still unclear, and there is no internationally recognized risk stratification scheme for ES patients^10^. Hence, it is urgent to determine the independent prognostic factors of ES and to accurately stratify ES patients’ risk.

Previous studies reported that ES’s potential clinical prognostic factors included age, race, tumour size, tumour stage and surgery^11,14,15^. However, these studies included a small number of predictors, which is prone to overfitting or biased fitting, resulting in different conclusions. We aim to combine different prognostic factors and establish a more accurate prognostic model based on larger and more recent samples. As a statistical prognostic model, nomograms are reliable and convenient and are widely used in oncology and medicine^15^. In this study, we extracted data from the Surveillance, Epidemiology, and End Results (SEER) database from 2010 to 2018 to determine the risk factors for overall survival (OS). A nomogram was established to quantify the survival rate of ES patients, and they were further categorized into three risk groups to predict their survival based on the total prognostic scores calculated by the nomogram^16^.

## Results

### Patient characteristics

The data of 1130 ES patients from 2010 to 2018 were extracted from the SEER database, of which 935 patients were included based on the inclusion and exclusion criteria. The primary cohort was randomly assigned to the training set (n = 656, 70%) and the validation set (n = 279, 30%). The clinicopathologic characteristics of patients in the training and validation sets are shown in Table 1 and Table S1.

**Table 1:** Multivariate Cox analysis of the training set on OS.

Variables	Patient no. (%)	OS
		HR (95% CI)
**Age (years)**		
≤ 18	397 (60.5)	Reference
19–34	163 (24.9)	1.67 (1.17–2.39)**
≥ 35	96 (14.6)	3.43 (1.84–6.42)***
**Site**		
Appendix	282 (43.0)	Reference
Axial	374 (57.0)	1.08 (0.79–1.47)
**Primary tumour number**		
1	599 (91.3)	Reference
≥ 2	57 (8.7)	1.22 (0.80–1.85)
**Tumour size (mm)**		
≤ 58	100 (15.2)	Reference
59–101	154 (23.5)	1.98 (1.18–3.34)*
≥ 102	100 (15.2)	1.64 (0.93–2.88)
Unknown	302 (46.1)	1.73 (1.03–2.94)*
**Lung metastasis**		
Yes	131 (20.0)	Reference
No/unknown	525 (80.0)	0.84 (0.57–1.25)
**Bone metastasis**		
Yes	116 (17.7)	Reference
No/unknown	540 (82.3)	0.66 (0.44–0.99)*
**Liver metastasis**		
Yes	11 (1.7)	Reference
No/unknown	645 (98.3)	0.49 (0.22–1.06)
**Tumour stage**		
Localized	198 (30.2)	Reference
Regional	205 (31.2)	1.35 (0.85–2.14)
Distant	253 (38.6)	2.33 (1.38–3.96)**
**Surgery**		
Yes	360 (54.9)	Reference
No/unknown	296 (45.1)	1.46 (1.03–2.07)*
**Chemotherapy**		
Yes	621 (94.7)	Reference
No/unknown	35 (5.3)	4.32 (2.52–7.41)***
**Radiotherapy**		
Yes	327 (49.8)	Reference
No/unknown	329 (50.2)	0.97 (0.71–1.33)
**Marital**		
Married/domestic partner	86 (13.1)	Reference
Single	542 (82.6)	0.97 (0.53–1.78)
Other	28 (4.3)	1.30 (0.70–2.41)

*OS* overall survival, *HR* hazard ratio, *CI* confidence interval, *Tumour* *stage* based on SEER Extent of Disease (EOD) following a SEER algorithm.

***p < 0.001, **p < 0.01, *p < 0.05.

### Survival analysis

Univariate analysis was used to calculate the effect of the included variables on survival outcomes, and the results are shown in Fig. S1. Sex (p = 0.9084), race (p = 0.3779) and brain metastasis (p = 0.9030) were not associated with significant differences in survival. Multiple Cox proportional hazard analysis for the remaining variables demonstrated that young patients (≤ 18 years old), small tumour size (≤ 58 mm), no/unknown bone metastasis, localized tumour stage, and received surgery and chemotherapy were independently linked with better survival, while race, sex, tumour site, the number of primary tumours, marital status, liver metastasis, lung metastasis, brain metastasis and radiotherapy were not associated with significant differences in survival. The results of the multivariate analysis are shown in Table 1 and Table S1.

### Nomogram construction and performance

**Figure 1:**
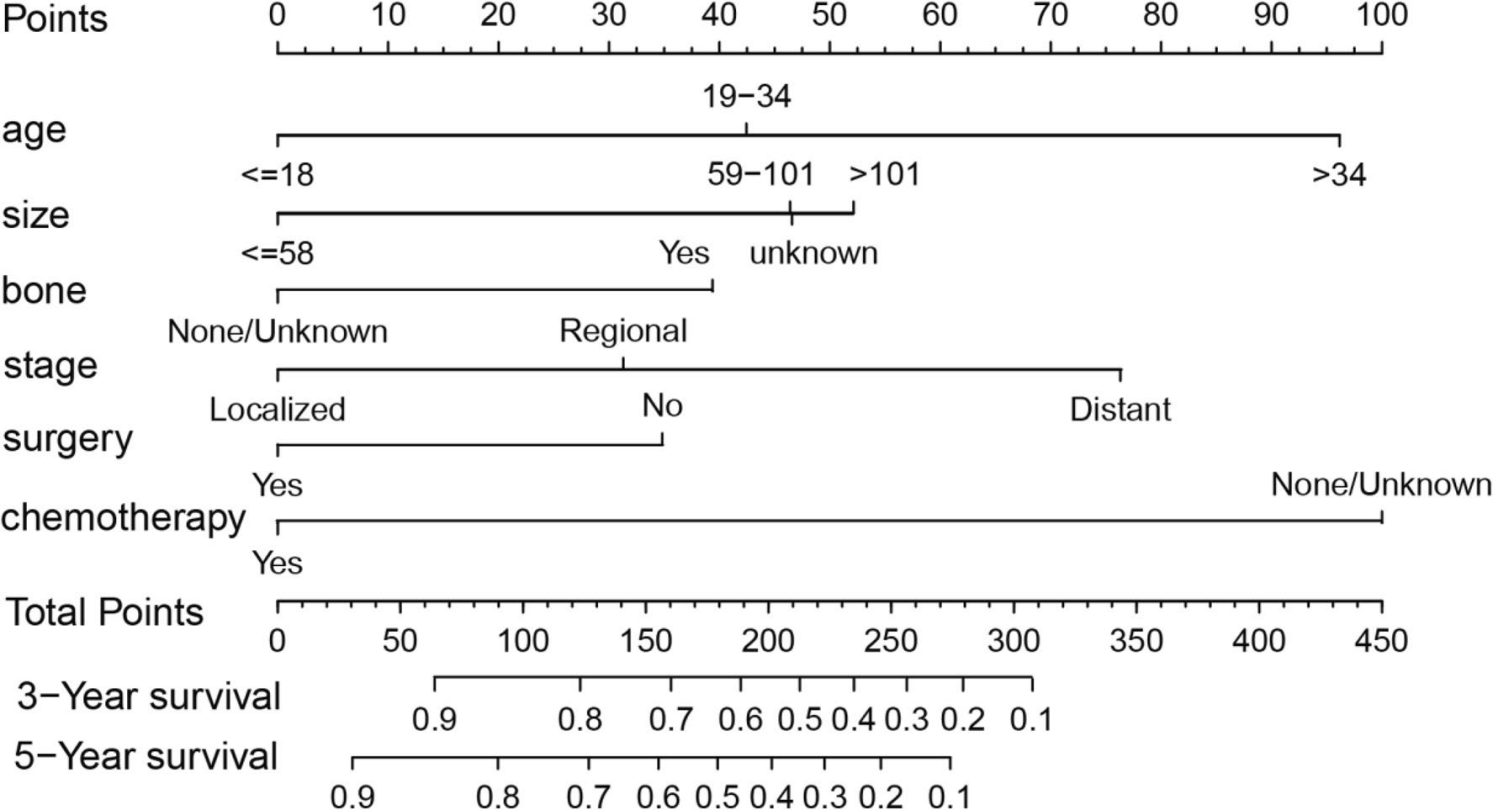
Nomogram for predicting the probability of 3- and 5-year survival based on OS.

The results of the multivariate analysis shown in Table 1 were utilized to construct the nomogram, which was subsequently used to generate individualized predictions of the 3- and 5-year OS of the ES patients (Fig. 1). The score scale calculation chart at the top was used to evaluate each prognostic factor, the total scores were added, and then, the 3- and 5-year OS were calculated as a guide. The weight of chemotherapy and age were the highest shown in the nomogram, followed by tumour stage and tumour size. None of the covariates were associated with time (Fig. S2). The PH assumption was met by OS (p = 0.20) models, as demonstrated by the Schönfeld test. Figure S3 shows that none of the individually observations were extremely influential.

**Figure 2:**
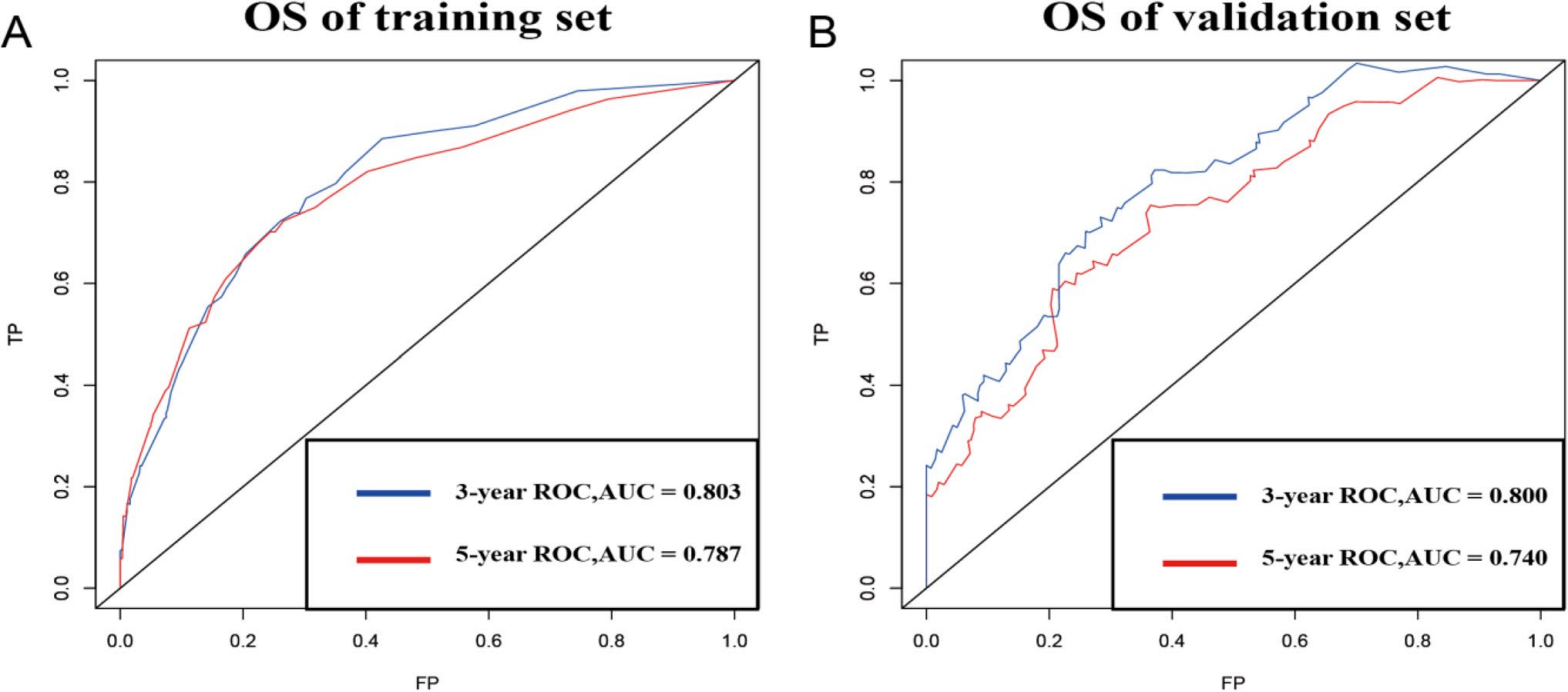
ROC. (**A**) ROC curves of the training set for 3- and 5-year survival. (**B**) Show the ROC of the validation set of 3- and 5-year survival.

**Figure 3:**
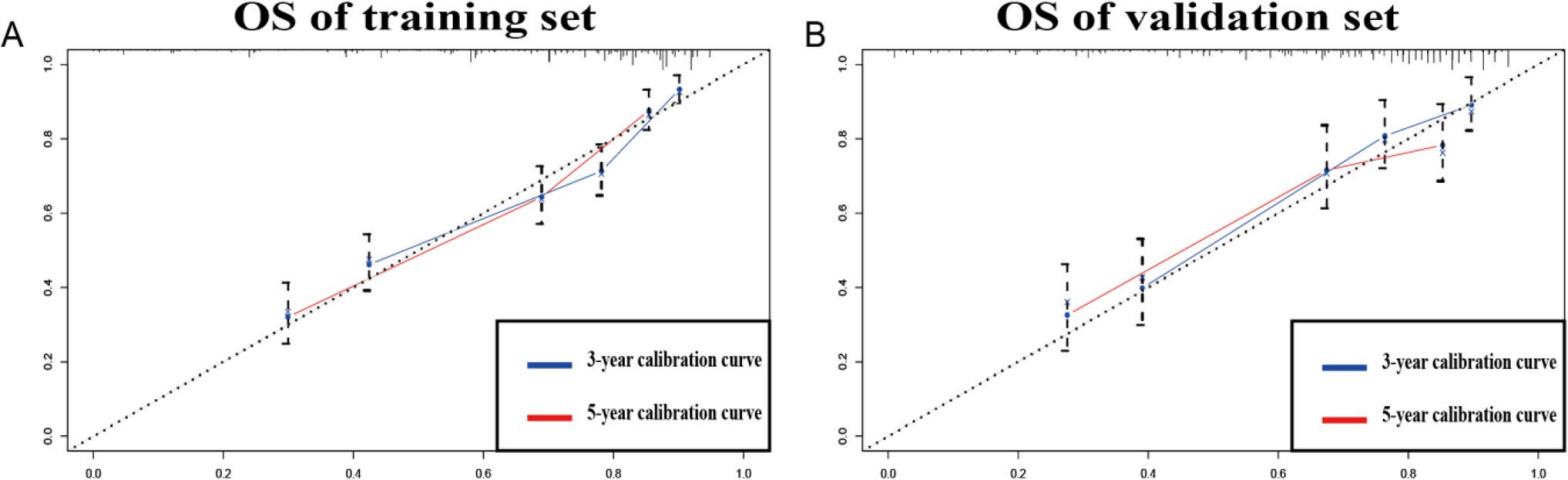
Calibration curves. (**A**) Calibration curves of the training set of 3- and 5-year survival. (**B**) Calibration curves of the validation set of 3- and 5-year survival.

The C-indices provided by the nomogram (validation and training sets) were higher than those of the SEER tumour staging system (OS: 0.788, 0.767 vs. 0.669, p < 0.01, p < 0.01), indicating that this multivariable model has higher discrimination for predicting the prognosis of ES. The area under the curve (AUC) values of the 3- and 5-year OS for the training and validation sets were 0.803 and 0.787 vs. 0.800 and 0.740, respectively (Fig. 2). The calibration curve, which is shown in Fig. 3 and indicates the predicted probabilities and observed outcomes of the model, demonstrated prominent accordance. The IDI and NRI results showed that the new model was better than SEER tumour stage in terms of predictive performance (Table 2).

**Table 2:** IDI and NRI of the nomogram on OS.

	Survival time	Items	Training set	Validation set
			Est (95% CI)	Est (95% CI)
OS	3-year	IDI	0.12 (0.06–0.17)***	0.13 (0.04–0.21)***
		NRI	0.33 (0.20–0.42)***	0.34 (0.13–0.45)***
	5-year	IDI	0.13 (0.07–0.19)***	0.09 (0.01–0.15)*
		NRI	0.30 (0.15–0.42)***	0.22 (0.04–0.37)*

*OS* overall survival, *Est* empower stats, *CI* confidence interval, *IDI* integrated discrimination improvement index, *NRI* category-less net reclassification index.

***p < 0.001, *p < 0.05.

### Stratifying the risk of patients

**Figure 4:**
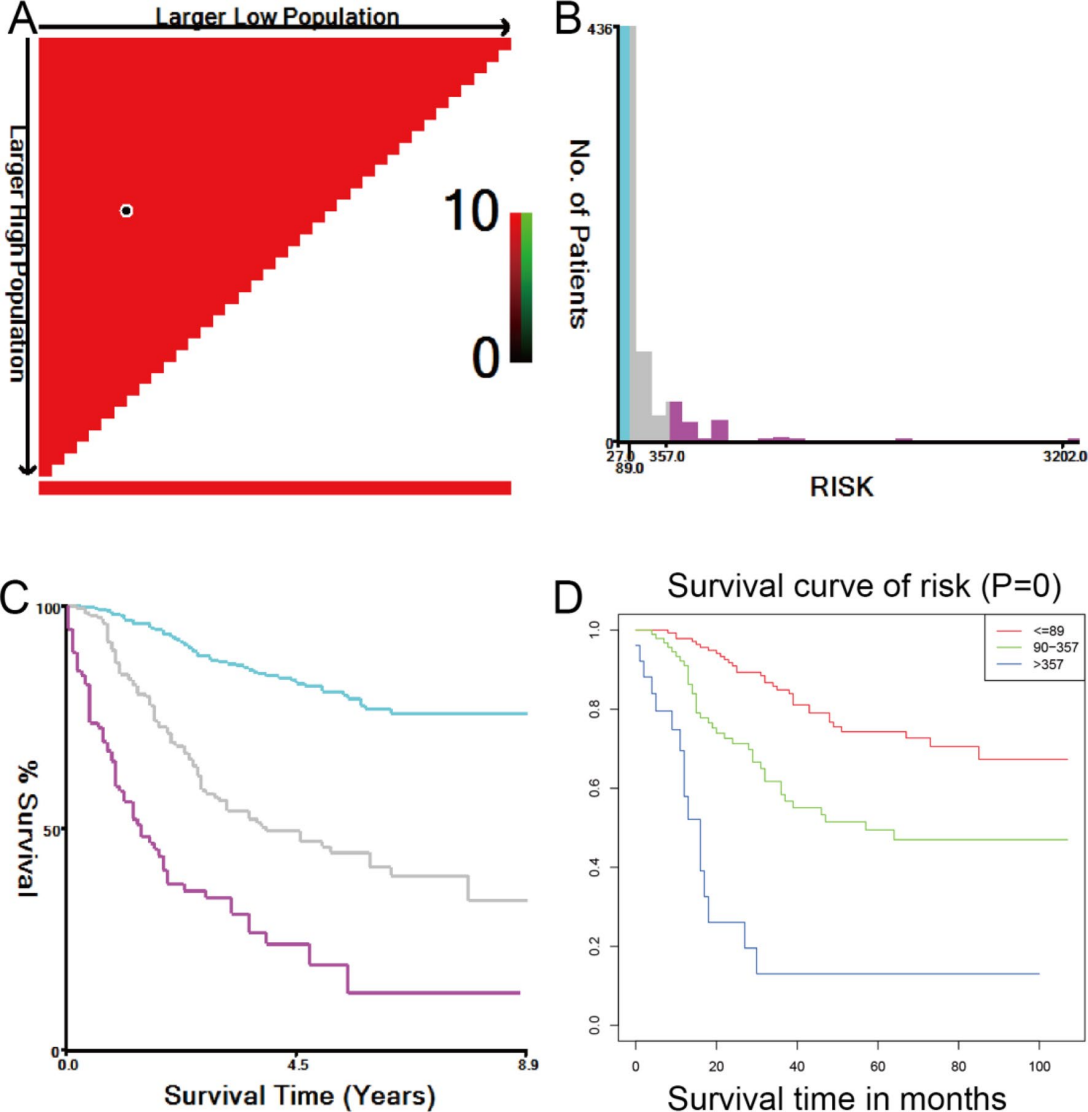
KM curves of the three risk groups of ES patients. (**A–C**) The best cut-off points of risk were defined via the X-tile program for the OS of the training set. (**D**) For the OS of the validation set.

Based on the nomogram, the total prognostic score of each patient was calculated, and patients in the training set were divided into three risk groups to estimate the probability of their OS in accordance with the cut-off points detected by X-tile (Fig. 4A–C). In the validation set, the Kaplan–Meier (KM) curves of OS demonstrated that the risk stratification was stable for predicting the probability of patient survival (Fig. 4D).

## Discussion

Ewing sarcoma is a highly malignant bone tumour with poor prognosis. However, due to its low incidence (less than 3 per 1,000,000), its prognostic factors are still controversial^17^. Current limitations among the different studies focus on the following three aspects: inadequate and old cases, the included variables were insufficient, and the model performed poorly and not validated fully. Hence, the conclusions vary widely between studies. A nomogram is a widely accepted prognostic model that integrates various prognostic factors to predict individual survival. This study extracted the latest ES data from 2010 to 2018 from the SEER database. The included variables in our study were based on previous studies to make the conclusions more representative. The prognostic factors were determined through univariate and multiple Cox proportional hazard analyses. Six independent prognostic factors were identified, and a nomogram was constructed to effectively and intuitively estimate 3- and 5-year OS. To the best of our knowledge, our nomogram provides an improved C-index compared with current studies and shows good discrimination and calibration.

Young age is generally considered to be associated with a better prognosis, while it has a higher incidence in people under the age of 30, especially in children and adolescents^18^. This might be because adult patients received few cases of chemotherapy, and older patients were more likely to have multiple comorbidities, including diabetes, high blood pressure, and secondary cancer^19^. Additionally, ES patients above 18 years old are more prone to metastasize at initial diagnosis^20^. A previous study reported that larger tumours were associated with the risk of recurrence and metastasis^21^. The metastasis risk can be up to triple with a tumour size greater than 118 mm^22^. Our research defined 58 mm and 101 mm as two cut-off points and found that tumour sizes between 59 and 101 mm were similar to unknown sizes. The results also showed that bone metastasis and tumour stage were important prognostic factors. Indeed, ES is an invasive type of tumour, with 25% of ES arising in soft tissues rather than bone, and approximately 20–32% of ES patients have distant metastasis^11,23^. The lungs are the most common site, followed by bone^24^. Interestingly, lung metastasis was not included in our nomogram, which might be because there was a potential correlation between distant tumour stage and lung metastasis. This further proved the importance of timely diagnosis. However, ES accounts for less than 1% of all cancer diagnoses each year, patients show delayed, nonspecific symptoms similar to common musculoskeletal injuries, and doctors usually have low suspicions^25^. It is worth noting that our research showed that surgery and chemotherapy were independently associated with OS, while radiotherapy was not found to be an independent prognostic factor. Previous studies reported that when there was no long-term risk of disability, surgery was usually recommended, chemotherapy was also a standard approach for initial treatment, and radiotherapy was only advised for inoperable lesions^19,26,27^. Through the application of multimodality approaches, the long-term survival rate of localized ES has improved by more than 50% over the last 30 years, while only 20% of ES patients with metastasis can survive for a long time^28^. Although the survival of ES patients has improved, only 55% of patients received appropriate therapy, which means many therapies were ineffective or unnecessary and hence led to serious late effects. For example, ES is considered radiation-sensitive, while radiotherapy has been controversial, and the proportion of patients who receive radiation alone has been steadily declining. This may be attributed to advances in orthopaedic surgery and chemotherapy and the late effects of radiation in children, such as second malignancies and growth disturbances^10^.

Our study also concluded that race, sex, tumour site, tumour number, marital status, brain and liver metastasis, and radiotherapy were not found to be independently associated with OS. In particular, race, sex and brain metastasis were excluded after the univariate analysis. Although ES is much more common in white populations and has a slight male predominance^29^, a previous study reported that ES patients’ morbidity and mortality were not closely related to race or sex^2^. Although it has been reported that axial tumours are more likely to metastasize at the time of diagnosis, the tumour site was not included in our study, which might be because it has a potential correlation with tumour stage^30,31^. The primary tumour number was seldom reported before, and we identified that it was not associated with ES, while the reason behind it remained unclear. Marital status was considered a nonindependent factor and may be related to age in ES, as ES is more common in children and adolescents^15,16^. Brain or liver metastasis was also not identified with a higher risk of death, possibly because few cases had metastasis to these two sites (less than 1.1%).

Finally, we divided the ES patients into three risk groups to predict their survival. As a small round cell malignant tumour, ES presents a similar morphology, which means it is difficult to distinguish the histology grade^32,33^. Additionally, owing to uniformly poor prognosis, there is no internationally recognized risk classification reference for patients with ES thus far^10^. Based on the nomogram, we developed a risk stratification scheme to predict the ES' OS and validated it with the validation set.

There were several limitations to this study. First, there was inevitably bias in retrospective studies, and large randomized controlled trials are needed. Second, there were several insufficient prognostic factors in the SEER database, such as genotype and tumour markers. Finally, data collected from other sources were deficient for external verification.

In conclusion, age at diagnosis, tumour size, bone metastasis, tumour stage, surgery and chemotherapy were identified as independent prognostic factors for ES. Based on these independent prognostic factors, a nomogram for OS was constructed. The nomogram provided an improved C-index compared with current studies and showed good discrimination and calibration. Based on the nomogram, ES patients were divided into three risk groups to predict their survival. More research is needed to determine whether it applies to other patient groups.

## Methods

### Data source and selection

Data from patients diagnosed with ES were extracted from the SEER database, which includes 18 population-based cancer registries covering 30% of the US population^34^. The SEER database does not provide case identification information, and patient consent is not required to use these data. The research methods were carried out in accordance with relevant guidelines and regulations. The data of ES patients were extracted according to the following criteria: (i) diagnosed with ES based on ICD-O-3 (Third Edition of the International Classification of Diseases for Oncology); (ii) histological confirmation; and (iii) patients with unknown tumour stage, tumour metastasis and race were excluded.

### Variables

**Figure 5:**
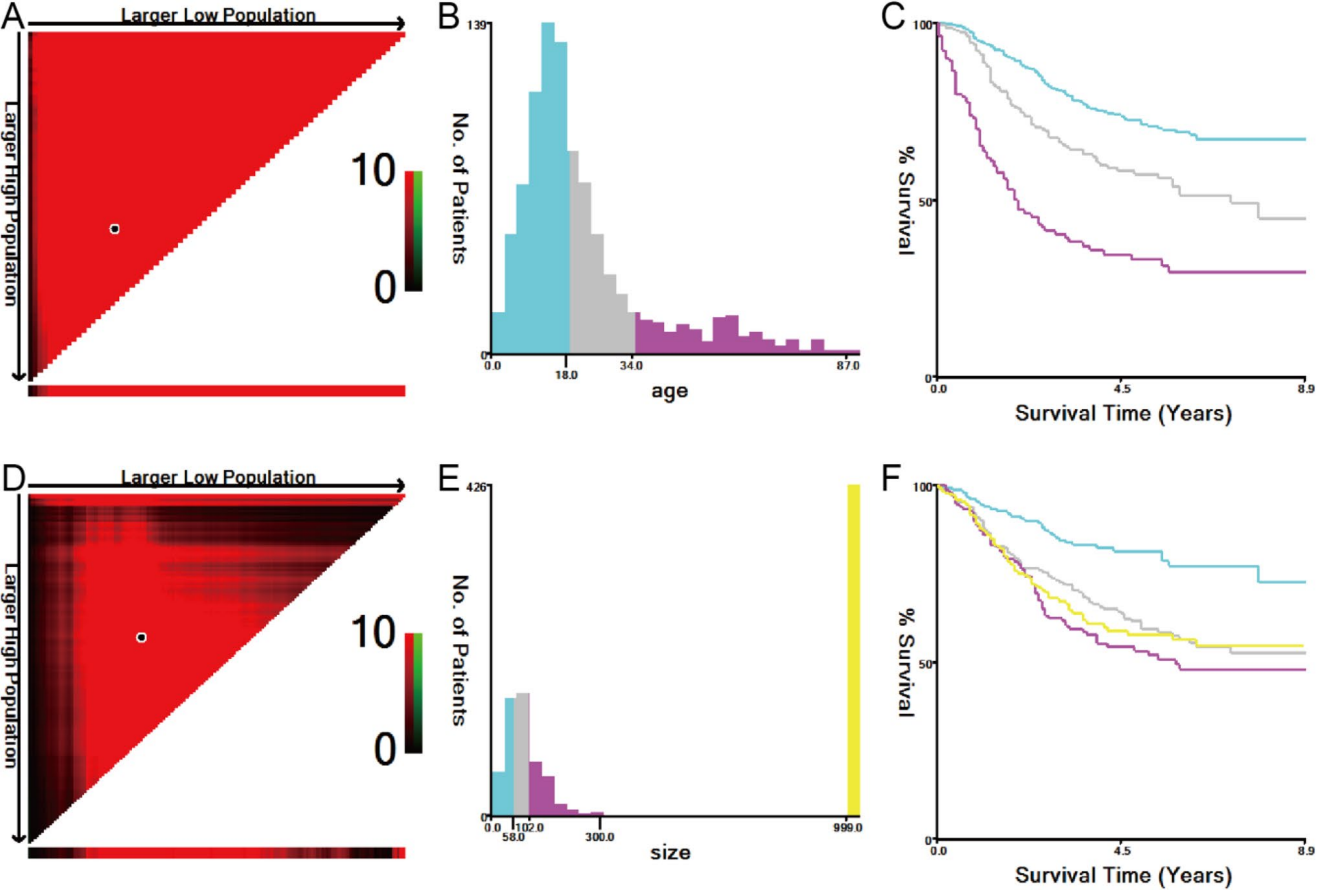
X-tile analysis of survival data. (**A–F**) The best cut-off points of age and tumour size were defined via the X-tile program. (**A,D**) The black dot indicates that optimal cut-off values have been identified. (**B,E**) A histogram and (**C,F**) KM curves were constructed based on the cut-off points.

The demographic variables of the patients who needed to be collected included age at diagnosis, race, sex, tumour site, number of primary tumours, marital status, tumour size, bone metastasis, brain metastasis, lung metastasis, liver metastasis, tumour stage (based on SEER Extent of Disease following a SEER algorithm), surgery, radiotherapy, chemotherapy, vital status and survival months. Age at diagnosis was stratified into three groups, while tumour size was stratified into four groups using the X-tile (Yale University, New Haven, CT, USA) program to obtain the best cut-off points (Fig. 5)^35^. The primary tumour site was divided into appendix (bones of limb and associated joints) and axial (mandible, vertebral column, rib, sternum, clavicle, pelvic bones, sacrum, coccyx and associated joints) regions. The tumour stage was divided into localized, regional, and distant. The tumour, which was confined entirely to the organ of origin, was defined as localized. Tumours that extended into surrounding organs or tissues were defined as regional. Tumours that spread to parts of the body remote from the primary tumour were defined as distant.

### Statistical analysis

Univariate and multiple Cox proportional hazard analyses were used to determine all independent risk factors, and a prognostic nomogram of OS for 3 and 5 years was constructed. The maximum score of each factor in the nomogram was 100 scores. By using the X-tile program, patients were divided into three risk groups based on the nomogram prognostic score, and their survival rates were predicted. The proportional hazards (PH) assumption was checked using statistical tests and graphical diagnostics based on the scaled Schoenfeld residuals. The deviance residuals were adopted to test influential observations. The concordance index (C-index), the receiver operating characteristic curve (ROC) and the calibration curve were used to analyse the capability of the nomogram. The calibration curve received 1,000 bootstrap repeats and then compared them with the actual survival time. In addition, by calculating the integrated discrimination improvement (IDI) and the net reclassification improvement (NRI), the model’s discrimination was compared with the SEER tumour stage.

Official SEER*Stat software (Version 8.3.9; NCI, Bethesda, USA) was used to collect data^36^. All statistical analyses were performed by R software version 4.11 (http://www.r-project.org/). The R packages used in this study included rms, survival, foreign, caret, survivalROC, survC1 and survIDINRI. A two-sided p value < 0.05 was considered statistically significant.

## Supplementary Information

Supplementary Legends.

Supplementary Figure S1.

Supplementary Figure S2.

Supplementary Figure S3.

Supplementary Table S1.
