# Supplementary material for: A novel nomogram and risk classification system predicting the Ewing sarcoma: a population-based study
Source: Sci Rep. 2022 May 17;12:8154. doi: 10.1038/s41598-022-11827-z (PMC9113999; doi:10.1038/s41598-022-11827-z)
Supplement: Supplementary file 1 — Supplementary Legends. [file 41598_2022_11827_MOESM1_ESM.docx]

Fig. S1: Kaplan–Meier curves estimated specific survival in patients with ES. The survival months of each group are represented by different colours. The variables were stratified by (A) age at diagnosis, (B) sex, (C) race, (D) marital status, (E) tumour site, (F) tumour size, (G) primary tumour number, (H) tumour stage, (I) metastasis to brain, (J) metastasis to liver, (K) metastasis to bone, (L) metastasis to lung, (M) surgery, (N) radiotherapy, and (O) chemotherapy.

Fig. S2: Graphs of the scaled Schoenfeld residuals against the transformed time based on OS.

The solid line is a smoothing spline fit to the plot, with the dashed lines representing a +/- 2-standard-error band around the fit. Systematic departures from a horizontal line are indicative of nonproportional hazards.

Fig. S3: Index plots of dfbeta for the Cox regression of time to death based on OS.

The plots comparing the magnitudes of the largest dfbeta values to the regression coefficients suggest that none of the observations is extremely influential individually.
