# Supplementary figures and images for: A novel nomogram and risk classification system predicting the Ewing sarcoma: a population-based study
Source: Sci Rep. 2022 May 17;12:8154. doi: 10.1038/s41598-022-11827-z (PMC9113999; doi:10.1038/s41598-022-11827-z)

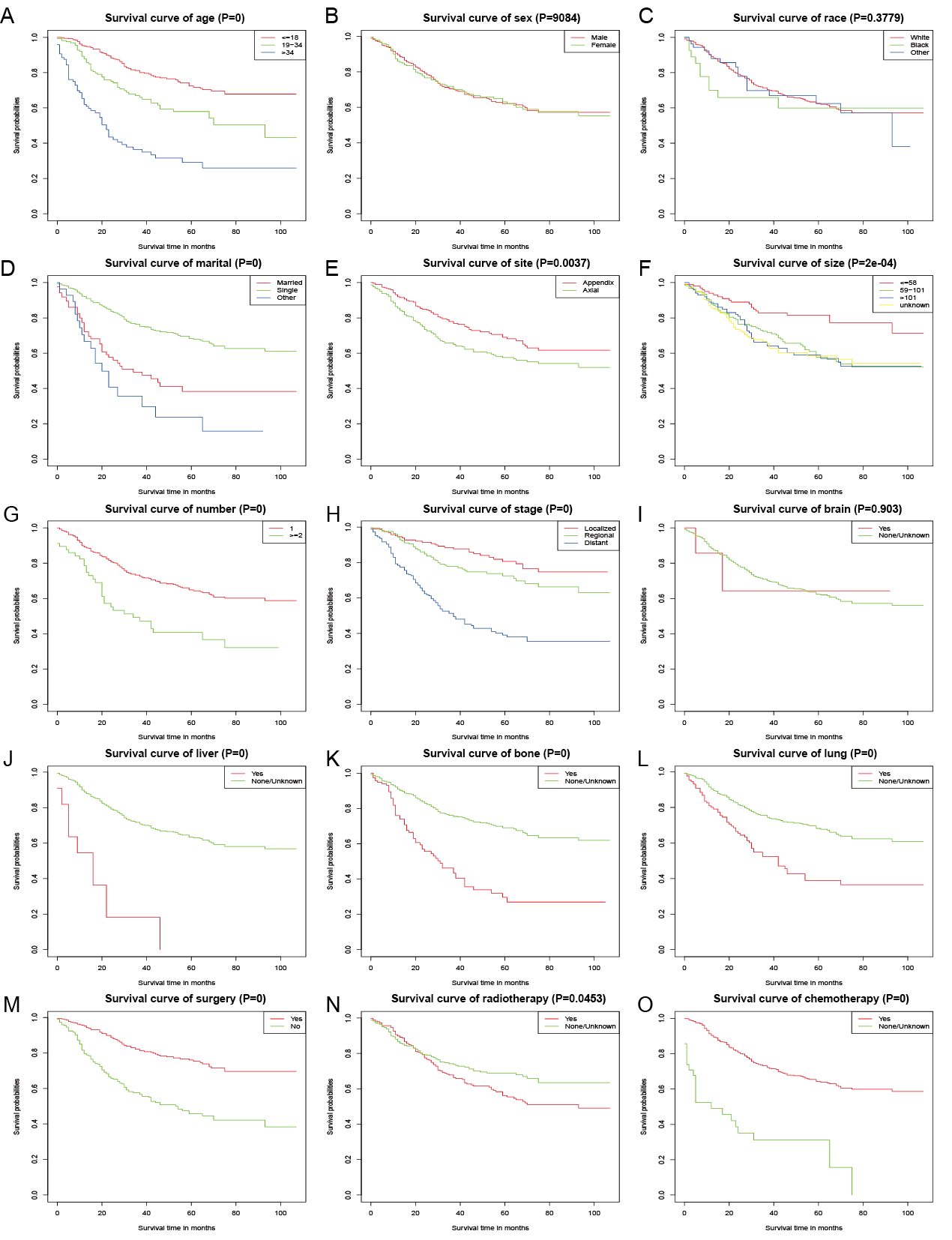

Supplement: Supplementary file 2 — Supplementary Figure S1. [file 41598_2022_11827_MOESM2_ESM.tif]

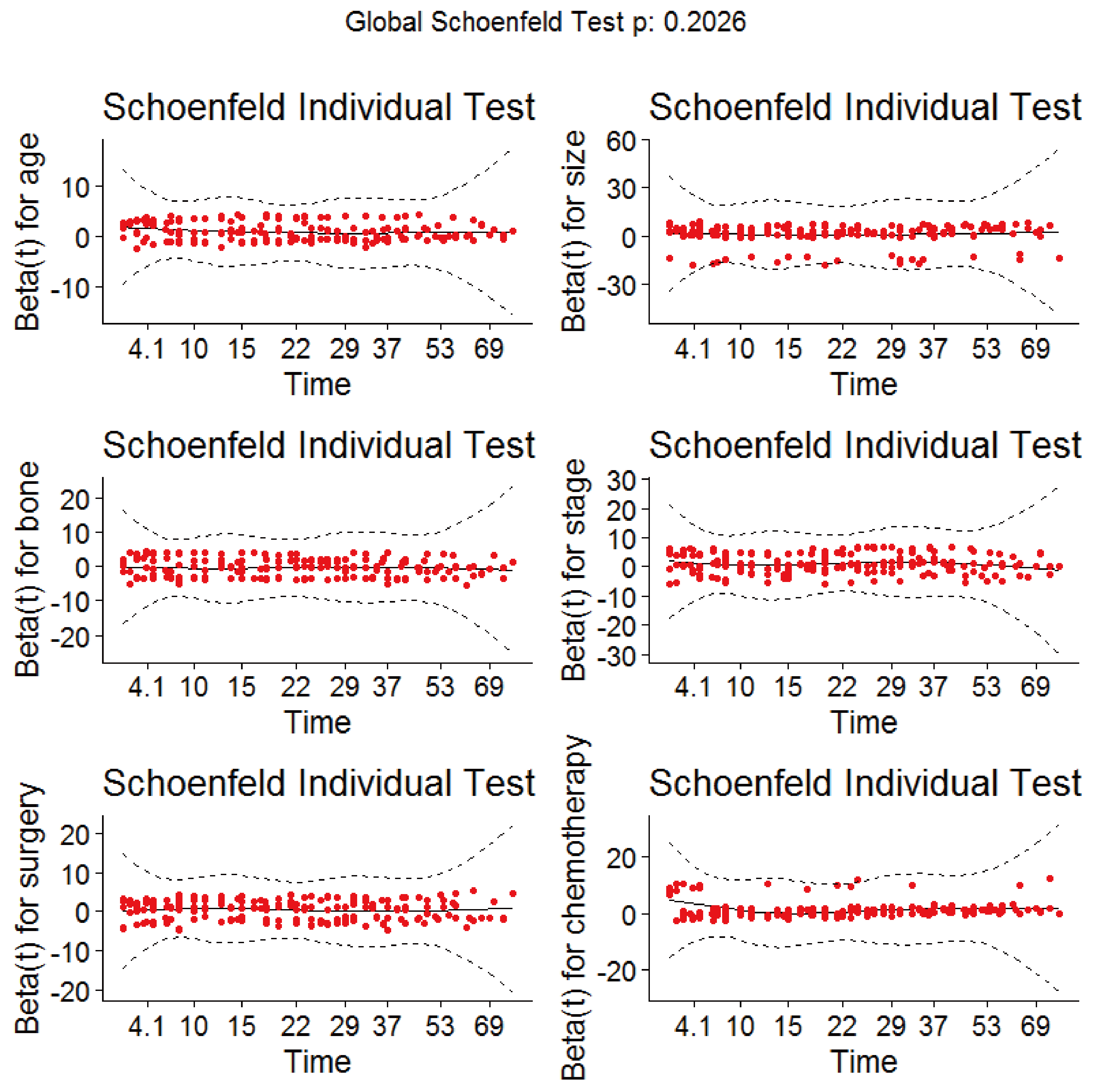

Supplement: Supplementary file 3 — Supplementary Figure S2. [file 41598_2022_11827_MOESM3_ESM.tif]

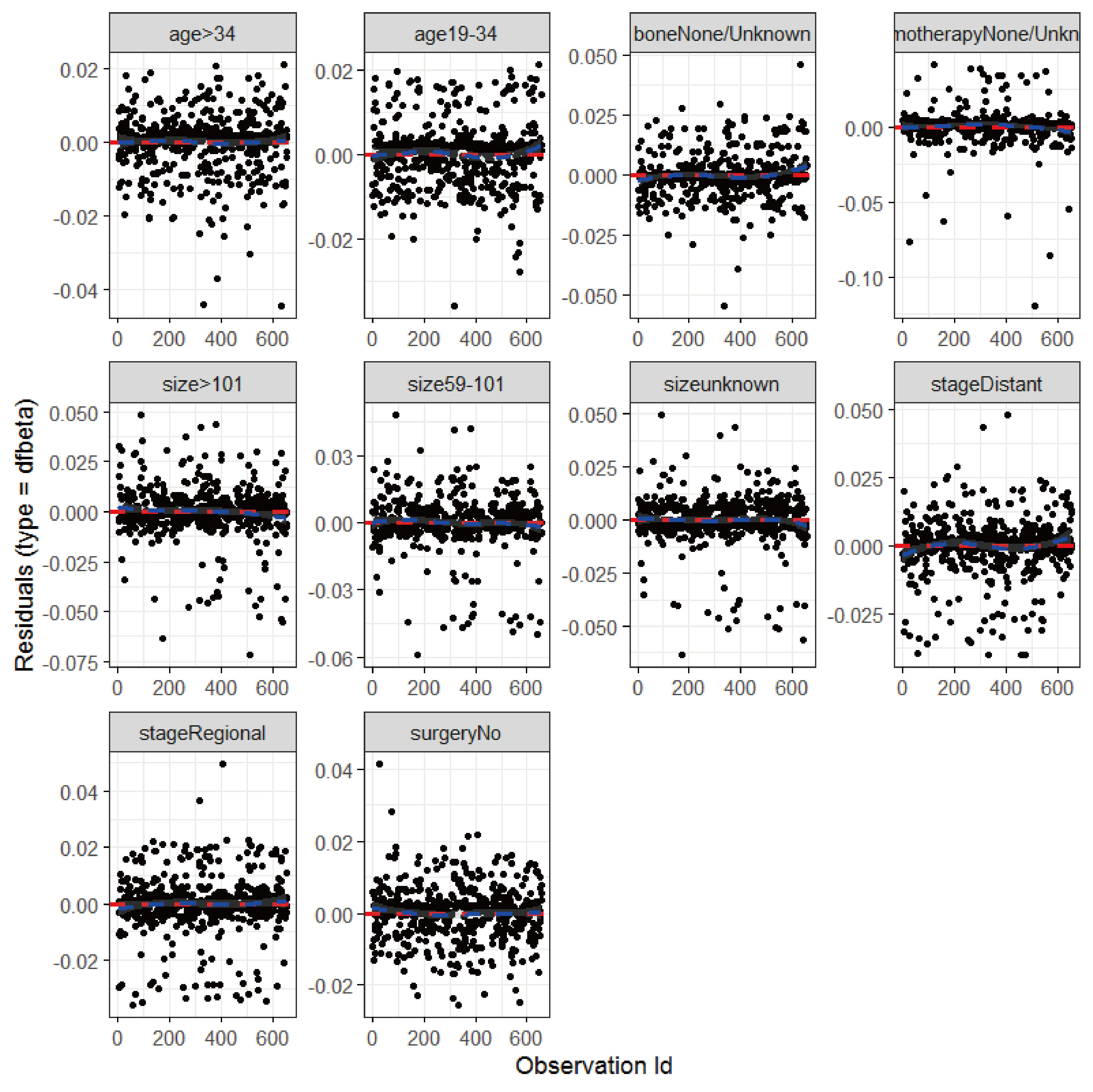

Supplement: Supplementary file 4 — Supplementary Figure S3. [file 41598_2022_11827_MOESM4_ESM.tif]
