# Supplementary material for: A novel nomogram and risk classification system predicting the Ewing sarcoma: a population-based study
Source: Sci Rep. 2022 May 17;12:8154. doi: 10.1038/s41598-022-11827-z (PMC9113999; doi:10.1038/s41598-022-11827-z)
Supplement: Supplementary file 5 — Supplementary Table S1. [file 41598_2022_11827_MOESM5_ESM.docx]

Table S1: Multivariate Cox analysis of the validation set on OS

| Variables | Patient no. (%) | OS |
| --- | --- | --- |
|  |  | HR (95% CI) |
| Age (years) |  |  |
| ≤18 | 157 (56.3) | Reference |
| 19-34 | 76 (27.2) | 1.27 (0.74-2.20) |
| ≥35 | 46 (16.5) | 4.27 (1.72-10.58) ** |
| Site |  |  |
| Appendix | 113 (40.5) | Reference |
| Axial | 166 (59.5) | 1.09 (0.66-1.79) |
| Primary tumor number |  |  |
| 1 | 254 (91.0) | Reference |
| ≥2 | 25 (9.0) | 1.09 (0.48-2.47) |
| Tumor size (mm) |  |  |
| ≤58 | 49 (17.6) | Reference |
| 59-101 | 57 (20.4) | 2.46 (1.06-5.74) * |
| ≥102 | 49 (17.6) | 4.57 (2.07-10.09) *** |
| Unknown | 124 (44.4)  （ | 1.92 (0.87-4.26) |
| Lung metastasis |  |  |
| Yes | 61 (21.9) | Reference |
| No/ Unknown | 218 (78.1) | 0.48 (0.23-0.98) * |
| Bone metastasis |  |  |
| Yes | 30 (10.8) | Reference |
| No/ Unknown | 249 (89.2) | 0.36 (0.18-0.74) ** |
| Liver metastasis |  |  |
| Yes | 2 (0.7) | Reference |
| No/ Unknown | 277 (99.3) | 0.57 (0.06-5.13) |
| Tumor stage |  |  |
| Localized | 93 (33.3) | Reference |
| Regional | 83 (29.8) | 1.17 (0.61-2.23) |
| Distant | 103 (36.9) | 1.04 (0.44-2.49) |
| Surgery |  |  |
| Yes | 171 (61.3) | Reference |
| No/Unknown | 108 (38.7) | 2.05 (1.23-3.41) ** |
| Chemotherapy |  |  |
| Yes | 268 (96.1) | Reference |
| No/Unknown | 11 (3.9) | 3.69 (1.35-10.09) * |
| Radiotherapy |  |  |
| Yes | 149 (53.4) | Reference |
| No/Unknown | 130 (46.6) | 0.93 (0.57-1.50) |
| Marital |  |  |
| Married/domestic partner | 48 (17.2) | Reference |
| Single | 221 (79.2) | 1.13 (0.78-2.64) |
| Other | 10 (3.6) | 1.38 (0.47-4.01) |

*** p <0.001, ** p < 0.01, * p <0.05

Abbreviations: OS, overall survival; HR: hazard ratio; CI: confidence interval; Tumour stage, based on SEER Extent of Disease (EOD) following a SEER algorithm.
